# *NFE2L2* polymorphisms, mortality, and metabolism in the general population

**DOI:** 10.1152/physiolgenomics.00178.2013

**Published:** 2014-05-01

**Authors:** Sylwia M. Figarska, Judith M. Vonk, H. Marike Boezen

**Affiliations:** Department of Epidemiology, University Medical Center Groningen, University of Groningen, Groningen, The Netherlands

**Keywords:** NFE2L2, all-cause mortality, general population, cardiovascular mortality, COPD mortality

## Abstract

The nuclear factor (erythroid-derived 2)-like 2 (*NFE2L2* or *NRF2*) gene regulates transcription of enzymes involved in cellular detoxification and lipids homeostasis. *NFE2L2* is associated with pathophysiology of atherosclerosis and chronic obstructive pulmonary disease (COPD). Therefore we studied the relation between *NFE2L2* and all-cause, cardiovascular, and COPD mortality and its associations with triglyceride and cholesterol levels. We genotyped five tagging single nucleotide polymorphisms (SNPs) (rs4243387, rs2364723, rs13001694, rs1806649, and rs6726395) in *NFE2L2* in 1,390 subjects from the Vlagtwedde-Vlaardingen cohort. Participants were examined in 1989/1990 and followed up till the vital status evaluation on December 31st, 2008. Associations between SNPs and mortality were estimated by Cox proportional hazards regression, and associations between SNPs and triglyceride and cholesterol levels were tested with linear regression. After 18 yr, 284 (20.4%) subjects had died, 107 from cardiovascular disease and 20 from COPD. Minor allele carriers of rs13001694 had a significantly reduced risk of all-cause mortality compared with wild types: hazard ratio (HR) 0.8 [95% confidence interval (CI) 0.6 to 1.0]. Minor allele carriers of rs2364723 had significantly reduced risk of cardiovascular mortality: HR = 0.5 (95% CI: 0.3–0.7). This result was consistent in stratified analyses: females 0.4 (0.2–0.7), males 0.6 (0.3–0.9), never smokers 0.5 (0.2–1.1), ever smokers 0.5 (0.3–0.8). Minor allele carriers of rs1806649 had a markedly reduced COPD mortality: HR = 0.3 (95% CI: 0.1–0.9). Rs2364723 was associated with lower triglyceride levels. None of the SNPs was associated with cholesterol levels. This study shows for the first time that *NFE2L2* is associated with reduced risk of all-cause, cardiovascular and COPD mortality in humans.

nearly 30% of the individual variance in life expectancy is genetically determined (6); however, the specific genetic determinants of the human lifespan still remain largely unknown. A candidate gene for predicting variation in human lifespan is the nuclear factor (erythroid-derived 2)-like 2 (*NFE2L2* or *NRF2*) gene. *NFE2L2* is a master regulator of antioxidant-related genes and genes that control immune and inflammatory responses and those involved in tissue remodeling (9) and thus has an important role in cytoprotection in the whole organism.

*NFE2L2* is a basic leucine zipper transcription factor and regulates expression of genes, via direct binding to the antioxidant responsive element in the target gene. The targets include genes encoding glutathione S-transferases, γ-glutamylcysteine ligases, heme oxygenase 1, and NADPH quinione oxidoreductase (4, 14). *NFE2L2* is expressed in all tissues, with highest levels in the key detoxification organs being the kidney and liver (11). Despite the well-documented relationship between *NFE2L2* and cellular protective mechanisms relevant to aging, very few studies have evaluated the role of *NFE2L2* in mediating rates of aging and longevity (10, 17, 22, 26). One of these studies indicated that *NFE2L2* may be important in longevity, since this gene was sixfold overexpressed in the liver of the naked mole rat, a species with a very long lifespan compared with mice, which have much shorter lifespans (26). In human fibroblasts it has been demonstrated that *NFE2L2* function declines in senescence, whereas silencing of *NFE2L2* leads to premature senescence (10). Furthermore, the broad role of *NFE2L2* in age-related diseases has been indicated by its association with the development of atherosclerosis (1, 5, 18, 27) and chronic obstructive pulmonary disease (COPD) (12, 15). Atherosclerosis is characterized by lipid deposition in the artery wall, and the impact of *NFE2L2* on atherosclerosis development may be mediated by its role in lipid homeostasis (8). The previous studies, performed in mice, have shown that the expression of *NFE2L2* regulates the expression of lipogenic genes and affects lipid accumulation and deposition in aortic lesions (1).

The Vlagtwedde-Vlaardingen cohort offers a unique opportunity to investigate the role of *NFE2L2* in long-term survival, since subjects included in this study were followed for 18 yr. Besides all-cause mortality, we evaluated the association between *NFE2L2* and cardiovascular and COPD mortality and we also tested the associations between SNPs in *NFE2L2* and triglyceride and cholesterol levels.

## METHODS

### 

#### Study population.

We studied subjects of the Vlagtwedde-Vlaardingen cohort, a general population-based cohort of exclusively Caucasian individuals of Dutch descent, recruited from Vlagtwedde, a rural area, and Vlaardingen, an urban area in the Netherlands (25). This cohort started in 1965, and participants had medical exams every 3 yr until the last survey in 1989/1990. In each survey the Dutch version of the British Medical Council standardized questionnaire was filled in, and spirometry was performed. In this study we included 1,390 subjects (16.4% of the original cohort) out of 2,467 subjects of whom DNA was collected in the final survey in 1989/1990 (those with DNA samples contained >1,500 ng isolated DNA). There were no differences in characteristics between the selected (*n* = 1,390) and nonselected group (25). The Vlagtwedde-Vlaardingen cohort was set up to study respiratory health in the general population. During *survey 2* and *3* (in 1970/72 and 1973/75) an add-on study was performed by cardiologists. At that time the prevalence of cardiovascular problems was much higher in males than in females, and therefore only males were included in this cardiovascular add-on study. In the surveys of 1970/72 and 1973/75, fasting serum triglyceride and total cholesterol levels were measured in 493 males out of the 1,390 genotyped subjects. The vital status of all participants in the Vlagtwedde-Vlaardingen study was assessed on December 31, 2008. We evaluated three mortality outcomes, i.e., all-cause mortality (excluding external causes of death) and cardiovascular and COPD mortality (either as primary or secondary cause of death). The causes of death were coded according to the International Classification of Diseases (ICD-9 and ICD-10, Table 1). Analyses on cause-specific mortality were performed at Statistics Netherlands (The Hague). The study protocol was approved by the local university medical hospital ethics committee, University of Groningen, University Medical Center Groningen, The Netherlands, and all participants gave their written informed consent.

**Table 1.** ICD codes for the investigated causes of death

Cause of Death	ICD-9	ICD-10
External causes*	≥ 800	S, T, V, W, X, Y
CVD	390–398, 401–405, 410–417, 420–438, 440–448, 451–459, 785.4	G45–G46, I00–I15, I20–I28, I30–I52, I60–I69, I70–I79, I80–I89, I95–I97, I98.2, I98.8, I99, M30–M31, N28.0, R02, R58
COPD	490–492, 494, 496	J40–J44, J47

* Suicides, homicides, traffic accidents, etc.

ICD, International Classification of Diseases; CVD cardiovascular disease; COPD, chronic obstructive pulmonary disease.

#### Sample collection, DNA extraction, and genotyping.

**Fig. 1.**
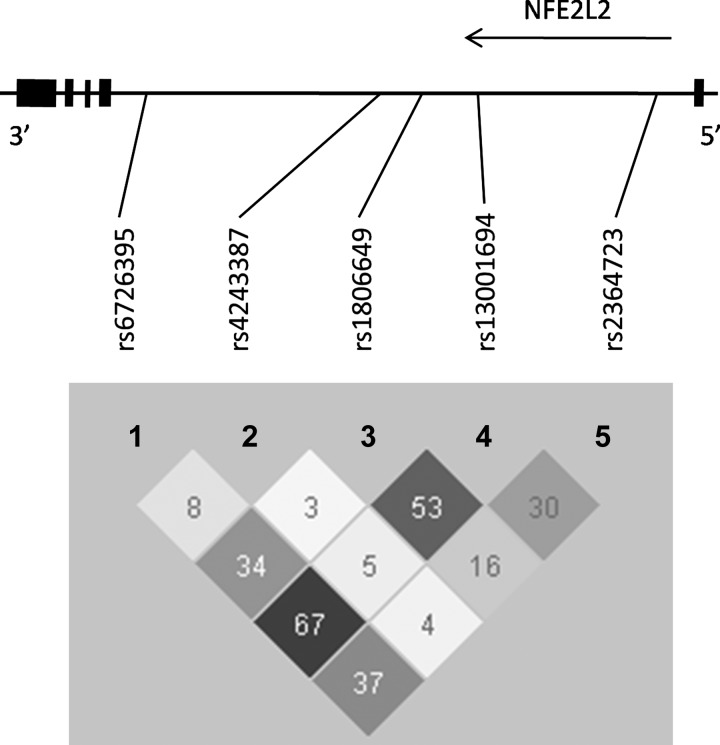
Position of genotyped single nucleotide polymorphisms (SNPs) in the nuclear factor (erythroid-derived 2)-like 2 (*NFE2L2*) gene and linkage disequilibrium plot (100·r^2^) in the Vlagtwedde-Vlaardingen cohort. The black boxes represent exons.

In 1989–1990, neutrophil depots from peripheral blood samples were collected and stored at −20°C. In 2003–2004 DNA was extracted from these samples with a QIAamp DNA blood mini kit (Qiagen, Hilden, Germany) and checked for purity and concentration with a NanoDrop ND-1000 UV-Vis spectrophotometer (NanoDrop Technologies, Wilmington, DE) (25). We pairwise tagged *NFE2L2* with five single nucleotide polymorphisms (SNPs) according to the HapMap CEU genotype data (release 23a) with r^2^ threshold of 0.8 and minor allele frequency >5%. SNPs were genotyped at K-Bioscience (UK) using their patent-protected competitive allele-specific PCR system (KASPar) (16). Figure 1 shows the position of genotyped SNPs in the *NFE2L2* gene and linkage disequilibrium (LD) between genotyped SNPs in the Vlagtwedde-Vlaardingen cohort. Hardy-Weinberg equilibrium was tested by the χ^2^-test (cut-off value *P* < 0.05).

#### Statistical analysis.

First, descriptive analyses were performed. Differences in genotype distribution between deceased and living subjects were tested by χ^2^-tests. Cox proportional hazards regression adjusted for sex, age, forced expiratory volume in 1 second (FEV_1_) %predicted, and pack-years of smoking (all at the 1989/1990 survey) was used to evaluate the association between SNPs and all-cause, cardiovascular, and COPD mortality. Time was defined from the examination in 1989/1990 until death, end of follow-up in 2008, or last registration if subjects were lost to follow-up. Survival curves were depicted based on these Cox models. In addition, stratified analyses according to sex and smoking habits were performed.

Linear regression adjusted for age at the measurement was used to evaluate the associations between lipid profile i.e., triglyceride and total cholesterol levels and SNPs. The triglyceride and cholesterol levels were logarithmically transformed to obtain a normal distribution. *P* values < 0.05 were considered statistically significant (tested 2-sided). All statistical analyses were performed with IBM SPSS 20 software.

## RESULTS

Table 2 shows the population characteristics at the survey in 1989/1990, according to vital status on December 31st, 2008. After 18 yr of follow-up, 78.2% (*n* = 1,087) of the cohort was still alive. We had an almost perfect follow-up, since only 19 (1.4%) of the genotyped participants were lost to follow-up. Among all 284 deaths, 107 (37.7%) occurred from cardiovascular disease (CVD), and 20 (14.2%) from COPD. The 14 (4.9%) deaths due to external causes were excluded from the analyses. All tested SNPs were in Hardy-Weinberg equilibrium. A comparison of the characteristics of the genotyped subjects at baseline (survey in 1989/90) stratified by genotype for each SNP (data not shown) shows that, except for a slightly higher FEV_1_% predicted in subjects homozygous for the minor allele of rs1806649, no differences between the genotypes were seen.

**Table 2.** Characteristics of participants at visit 1989/1990 by vital status on Dec. 31st, 2008

Status on 31 Dec. 2008	Alive	Deceased	*P* Value
*n* (%)	1,087 (78.2)	284 (20.4)	
Men	525 (48.3)	178 (62.7)	<0.001
Vlagtwedde	981 (90.2)	258 (90.8)	0.761
Age at last status, yr	68.4 (54.2–90.8)	72.6 (41.4–87.4)	<0.001
Ever smokers	711 (65.4)	219 (77.1)	<0.001
Pack-years in ever smokers	17.2 (0.1–117.1)	27.1 (0.6–262.2)	<0.001
FEV_1_ % predicted	94.6 (13.9)	84.3 (18.0)	<0.001
Causes of death			
CVD*		107 (37.7)	
COPD*		20 (7.0)	
External causes		14 (4.9)	
Other causes		149 (52.5)	

All variables are expressed as number (%) or mean (SD) or median (range) as appropriate. FEV_1_, forced expiratory volume in 1 second.

* Either as primary or secondary cause of death: 6 subjects had both CVD and COPD as primary and secondary cause of death.

### 

#### All-cause, cardiovascular, and COPD mortality.

**Fig. 2.**
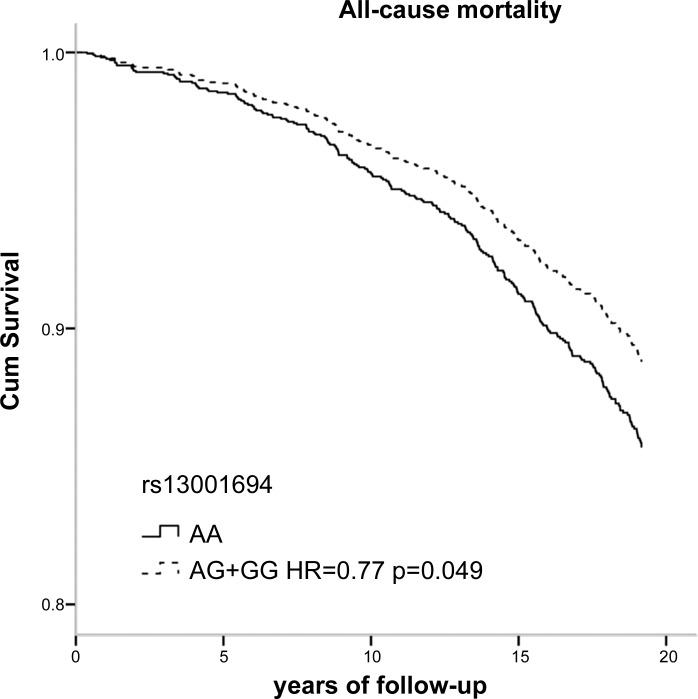
Survival curves for all-cause mortality according to SNP rs13001694. Cum, cumulative; HR, hazard ratio.

**Fig. 3.**
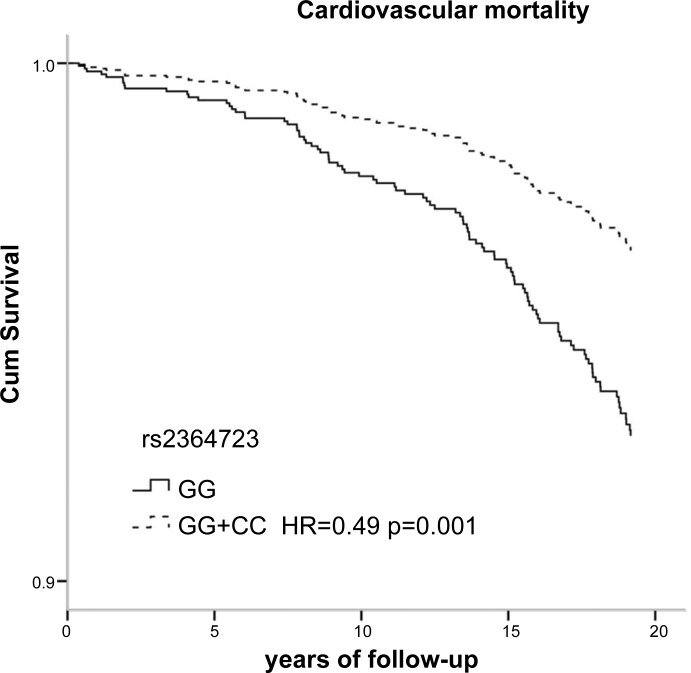
Survival curves for cardiovascular mortality according to SNP rs2364723.

**Fig. 4.**
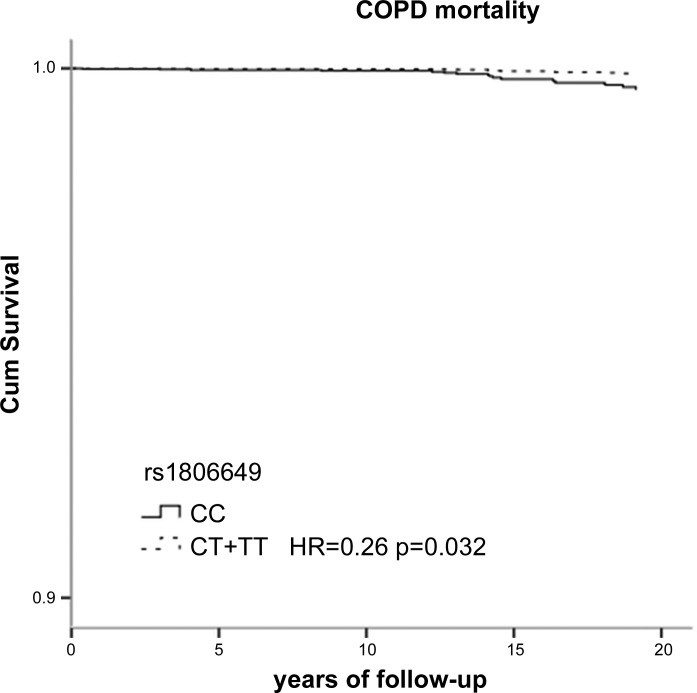
Survival curves for chronic obstructive pulmonary disease (COPD) mortality according to SNP rs1806649.

Table 3 shows the genotype distributions and the hazard ratios (HR) of living subjects and those who had died during 18 yr of follow-up. Among subjects who died, the carriers of the minor allele of SNP rs13001694 were significantly less common (58%) than in living subjects (66%). Furthermore, individuals carrying the minor allele of rs13001694 had a significantly reduced HR for all-cause mortality compared with wild types [HR 0.77; 95% confidence interval (CI), 0.59 to 1.00; *P* = 0.049] (Table 3). Stratified analysis according to sex showed no significant HR in females and males [0.83 (0.54–1.28) and 0.77 (0.55–1.07), respectively] (Table 4). The stratified analysis according to smoking habits showed that the protective effect of rs13001694 was observed in ever smokers only: HR = 0.71 [95% CI (0.53–0.95), *P* = 0.023] (Table 5). Carriers of the minor allele of rs2364723 had a significantly reduced risk of cardiovascular mortality: HR = 0.49, (0.33–0.74). In stratified analyses the risk of cardiovascular mortality associated with SNP rs2364723 was shown to be robust: females 0.35 (0.17–0.70), males 0.56 (0.33–0.93), never smokers 0.46 (0.20–1.07), ever smokers 0.50 (0.31–0.79), (Tables 4 and 5). Carriers of the minor allele of rs1806649 had a reduced risk of COPD mortality: HR = 0.26 (0.08–0.89). Survival curves according to genotypes of rs13001694, rs2364723, and rs1806649 clearly show the differences in mortality risk (Figs. 2, 3, and 4, respectively). Dominant genetic models (homozygous wild-type individuals vs. heterozygous/homozygous minor allele individuals) were used for all analyses, since the number of individuals homozygous for the minor allele was low, especially within subjects who died from CVD or COPD. Results of codominant models (data not shown) indicate that, in general, the HRs for heterozygotes and homozygotes for the minor allele are similar.

**Table 3.** Distribution of genotypes and HR for all-cause, CVD, and COPD mortality

SNP	Genotype	Alive	Deceased	CVD Deceased	COPD Deceased	All-cause Mortality HR (95% CI)	Cardiovascular Mortality HR (95% CI)	COPD Mortality HR (95% CI)
rs4243387	TT	898 (85.0)	238 (85.3)	84 (80.0)	19 (95.0)			
	TC+CC	158 (15.0)	41 (14.7)	21 (20.0)	1 (5.0)	0.96 (0.67–1.37)	1.52 (0.93–2.47)	0.42 (0.06–3.23)
rs2364723	GG	481 (46.1)	117 (41.9)	60 (56.6)	6 (30.0)			
	GC+CC	562 (53.9)	162 (58.1)	46 (43.4)*	14 (70.0)	0.96 (0.74–1.25)	0.49 (0.33–0.74)*	1.46 (0.45–4.72)
rs13001694	AA	354 (33.7)	117 (42.4)*	40 (38.5)	9 (45.0)			
	AG+GG	697 (66.3)	159 (57.6)	64 (61.5)	11 (55.0)	0.77 (0.59–1.00)*	0.89 (0.58–1.34)	0.47 (0.18–1.24)
rs1806649	CC	549 (53.8)	153 (57.5)	55 (54.5)	12 (66.7)			
	CT+TT	471 (46.2)	113 (42.5)	46 (45.5)	6 (33.3)	0.94 (0.72–1.22)	0.96 (0.63–1.46)	0.26 (0.08–0.89)*
rs6726395	GG	305 (28.9)	95 (34.4)	29 (27.4)	9 (45.0)			
	GA+AA	751 (71.1)	181 (65.6)	77 (72.6)	11 (55.0)	0.89 (0.67–1.17)	1.23 (0.78–1.94)	0.48 (0.18–1.28)

HR, hazard ratio; CI, confidence interval.

* *P* < 0.05.

**Table 4.** Distribution of genotypes and HR for all-cause and cardiovascular mortality stratified according to sex

		Women					Men				
SNP	Genotype	Alive	Deceased	CVD Deceased	All-cause Mortality HR (95% CI)	CVD Mortality HR (95% CI)	Alive	Deceased	CVD Deceased	All-cause Mortality HR (95% CI)	CVD Mortality HR (95% CI)
rs4243387	TT	467 (86.5)	93 (88.6)	33 (82.5)			431 (83.5)	145 (83.3)	51 (78.5)		
	TC+CC	73 (13.5)	12 (11.4)	7 (17.5)	0.96 (0.51–1.81)	1.71 (0.75–3.91)	85 (16.5)	29 (16.7)	14 (21.5)	0.98 (0.63–1.51)	1.47 (0.81–2.70)
rs2364723	GG	257 (47.9)	44 (41.9)	26 (65.0)			224 (44.2)	73 (42.0)	34 (51.5)		
	GC+CC	279 (52.1)	61 (58.1)	14 (35.0)*	0.91 (0.59-1.39)	0.35 (0.17-0.70)	283 (55.8)	101 (58.0)	32 (48.5)	0.95 (0.68–1.33)	0.56 (0.33–0.93)
rs13001694	AA	180 (33.1)	40 (39.2)	12 (31.6)			174 (34.3)	77 (44.3)	28 (42.4)		
	AG+GG	364 (66.9)	62 (60.8)	26 (68.4)	0.83 (0.54-1.28)	1.25 (0.60-2.61)	333 (65.7)	97 (55.7)*	38 (57.6)	0.77 (0.55-1.07)	0.78 (0.47-1.31)
rs1806649	CC	290 (54.7)	54 (54.0)	20 (52.6)			259 (52.9)	99 (59.6)	35 (55.6)		
	CT+TT	240 (45.3)	46 (46.0)	18 (47.4)	1.25 (0.82–1.92)	1.23 (0.62–2.44)	231 (47.1)	67 (40.4)	28 (44.4)	0.82 (0.58–1.15)	0.89 (0.53–1.50)
rs6726395	GG	162 (29.5)	35 (34.0)	10 (25.0)			143 (28.2)	60 (34.7)	19 (28.8)		
	GA+AA	387 (70.5)	68 (66.0)	30 (75.0)	0.86 (0.55–1.33)	1.44 (0.67–3.09)	364 (71.8)	113 (65.3)	47 (71.2)	0.93 (0.65–1.32)	1.17 (0.66–2.07)

SNP, single nucleotide polymorphism.

* *P* < 0.05.

**Table 5.** Distribution of genotypes and HR for all-cause and cardiovascular mortality stratified according to smoking habits

		Never Smokers					Ever Smokers				
SNP	Genotype	Alive	Deceased	CVD Deceased	All-cause Mortality HR (95% CI)	CVD Mortality HR (95% CI)	Alive	Deceased	CVD Deceased	All-cause Mortality HR (95% CI)	CVD Mortality HR (95% CI)
rs4243387	TT	313 (87.2)	55 (87.3)	21 (84.0)			585 (83.9)	183 (84.7)	63 (78.8)		
	TC+CC	46 (12.8)	8 (12.7)	4 (16.0)	0.93 (0.40–2.19)	1.59 (0.53–4.71)	112 (16.1)	33 (15.3)	17 (21.2)	0.99 (0.66–1.47)	1.53 (0.89–2.66)
rs2364723	GG	161 (45.2)	29 (45.3)	16 (61.5)			320 (46.6)	88 (40.9)	44 (55.0)		
	GC+CC	195 (54.8)	35 (54.7)	10 (38.5)	0.88 (0.51–1.53)	0.46 (0.20–1.07)	367 (53.4)	127 (59.1)	36 (45.0)	0.96 (0.71–1.29)	0.50 (0.31–0.79)*
rs13001694	AA	128 (35.9)	18 (28.6)	6 (25.0)			226 (32.6)	99 (46.5)	34 (42.5)		
	AG+GG	229 (64.1)	45 (71.4)	18 (75.0)	1.10 (0.61–2.00)	1.57 (0.57–4.33)	468 (67.4)	114 (53.5)*	46 (57.5)	0.71 (0.53–0.95)*	0.80 (0.50–1.27)
rs1806649	CC	199 (56.2)	32 (51.6)	12 (48.0)			350 (52.6)	121 (59.3)	43 (56.6)		
	CT+TT	155 (43.8)	30 (48.4)	13 (52.0)	1.47 (0.84–2.55)	1.44 (0.62–3.33)	316 (47.4)	83 (40.7)	33 (43.4)	0.84 (0.62–1.14)	0.86 (0.53–1.39)
rs6726395	GG	112 (30.9)	16 (25.8)	6 (23.1)			193 (27.8)	79 (36.9)	23 (28.8)		
	GA+AA	250 (69.1)	46 (74.2)	20 (76.9)	1.12 (0.60–2.09)	1.49 (0.55–4.04)	501 (72.2)	135 (63.1)*	57 (71.2)	0.85 (0.62–1.15)	1.19 (0.71–2.00)

* *P* < 0.05.

#### Triglycerides and cholesterol level.

Heterozygotes and homozygotes for the minor allele of rs2364723 had 0.084 lower ln(triglyceride levels) compared with the wild types, whereas heterozygotes and homozygotes for the minor allele of rs13001694, rs1806649, and rs6726395 had respectively 0.110, 0.113, and 0.107 higher ln(triglyceride levels) compared with their wild types.

None of the SNPs was significantly associated with cholesterol levels (Table 6). Results of codominant models (data not shown) indicate that, in general, the effect estimates for heterozygotes and homozygotes for the minor allele are comparable.

**Table 6.** Estimated effects of the NFE2L2 genetic variation on Ln-transformed triglyceride and cholesterol levels

		Triglyceride Levels				Cholesterol Levels			
SNP	Genotype	*n*	B	SE	*P* Value	*n*	B	SE	*P* Value
rs4243387	TT	400				345			
	TC+CC	83	0.015	0.056	0.783	76	−0.020	0.023	0.397
rs2364723	GG	218				184			
	GC+CC	259	−0.084	0.043	0.050	231	−0.006	0.018	0.756
rs13001694	AA	176				164			
	AG+GG	299	0.110	0.044	0.013	249	0.026	0.019	0.172
rs1806649	CC	254				227			
	CT+TT	205	0.113	0.043	0.009	174	0.003	0.019	0.875
rs6726395	GG	142				130			
	GA+AA	336	0.107	0.047	0.022	288	0.014	0.020	0.486

* Regression coefficient (B), its standard error (SE), and *P* value obtained with linear regression analysis adjusted for age at the measurement.

Cigarette smoking has an adverse effect on blood lipids, and, indeed in the Vlagtwedde-Vlaardingen cohort, smoking (pack-years) was associated with triglyceride and cholesterol levels. However, smoking does not seem to be a confounder of the associations between lipids and SNPs, since in the analysis additionally adjusted for smoking (pack-years) the effects of the SNPs on triglycerides and cholesterol levels remained similar (data not shown).

## DISCUSSION

We found a 20% reduced mortality risk among minor allele carriers of SNP rs13001694 in *NFE2L2* during the 18 yr of follow-up in the general population. Recently, a study that compared expression of oxidoreduction genes in the naked mole rat, the longest-living rodent known, to expression in mice, has shown that *NFE2L2* was sixfold higher expressed in the rats (26). Here we show evidence to support the hypothesis that *NFE2L2* may be one of the genes contributing to individual differences in human lifespan. In further analysis of survival in the Vlagtwedde-Vlaardingen cohort, we showed that the effect is independent of sex, since the effect was similar in females and males. In stratification according to smoking habits we found the significant protective effect of rs13001694 in ever smokers only, so it seems that the effect of the A/G substitution in rs13001694 may be exerted only under oxidative stress conditions. Indeed, a previous study showed that cigarette smoke exposure, a potent source of oxidative stress in the human lungs (3), may influence *NFE2L2* activity in alveolar macrophages by inducing *NFE2L2* nuclear accumulation and upregulation of *NFE2L2* target genes (19). Furthermore, SNP rs10183914, which is highly correlated with rs13001694 (r^2^ = 0.97, 1000 Genomes project), showed a protective effect on lung function decline in smokers in the Lung Health Study (LHS) (15). However, this result was not replicated when rs13001694 was tested in smokers from the Vlagtwedde-Vlaardingen cohort (15). Sandford et al. (15) explain that the lack of replication may be due to differences in recruitment between the two studies, as the LHS selected mild to moderate COPD patients and the Vlagtwedde-Vlaardingen cohort was from the general population, and the genetic factors associated with lung function decline in COPD patients and in the general population could be different. In our study, we adjusted the analysis for FEV_1_% predicted, thus the association of rs13001694 with better survival is independent of lung function level.

Another important finding in our study is that SNP rs2364723 is associated with a reduced risk of cardiovascular mortality. Even more interesting, the protective effect of this SNP was consistent within groups that have different mortality risks, i.e., females and males and never and ever smokers. This finding is of high importance since CVD is a main cause of morbidity and a leading cause of death in the elderly. A previous study in the Vlagtwedde-Vlaardingen cohort on the *NFE2L2* gene investigated its relationship to lung function level and decline (16). Interestingly, SNP rs2364723 was associated with a lower FEV_1_ level, and the same direction of the effect was observed in the British 1958 Birth Cohort (7, 16). Based on our finding showing the protective effect of this SNP on cardiovascular mortality, different pathways via which *NFE2L2* acts in pulmonary and cardiovascular events might be involved. There is evidence that *NFE2L2* plays a key role in preserving a healthy endothelial phenotype and maintaining the functional integrity of vasculature (24). Furthermore, *NFE2L2* dysfunction may lead to functional impairment of arteries, increasing susceptibility of blood vessels to injury in metabolic diseases (23). *NFE2L2* is essential for normal endothelial angiogenic processes, and its dysfunction may be a potential mechanism underlying impaired angiogenesis and decreased microvascular density in aging (24). Several studies suggest that *NFE2L2* alters susceptibility to atherosclerosis (1, 5, 18, 27). One of these studies indicated that activation of *NFE2L2* may exert antiatherogenic effects in vascular endothelium by suppressing inflammation (27), whereas another showed *NFE2L2* expression unexpectedly promotes atherosclerotic lesions formation (1). *NFE2L2* may affect atherosclerosis development since it regulates hepatic lipid homeostasis via activation of lipogenic genes expression (1, 8, 21). We found that rs2364723 was associated with lower triglyceride levels in male subjects. Triglyceride level is highly related to cardiovascular mortality; therefore, it is plausible that rs2364723 contributes to the observed, reduced cardiovascular mortality via its favorable effect on triglyceride levels. The publicly available data of the British 1958 Birth Cohort (7) showed that rs2364723 is also associated with lower cholesterol levels, and it gives us another intermediate phenotype by which rs2364723 exerts its overall protective effect on cardiovascular mortality. This SNP is in LD (r^2^ = 0.99) with promoter polymorphism rs35652124 (−653A/G) in the Vlagtwedde-Vlaardingen cohort (16). Marzec et al. (13) have reported that rs35652124 impedes the transcriptional activity of *NFE2L2* in vitro. Hence, it is possible that rs2364723 is not the causative variant, but that its association with cardiovascular mortality, due to almost complete LD, represents the effect of rs35652124. *NFE2L2* knockout mice exhibit ∼50% reduction in the degree of aortic atherosclerosis compared with the wild-type littermates (1); thus in this light a SNP that attenuates *NFE2L2* expression or activity is indeed likely to have the protective potential for cardiovascular morbidity and mortality in humans. Furthermore, it is known that decreased expression of *NFE2L2* in vitro is associated with acute lung injury, characterized by pulmonary edema and inflammation (13). Therefore, it seems that *NFE2L2* may have different local vascular and pulmonary effects, which could explain the opposite effects of rs2364723 on cardiovascular mortality and triglyceride levels on the one hand and on lung function level (FEV_1_) on the other hand.

The other investigated SNPs (rs13001694, rs1806649, and rs6726395) were associated with increased triglyceride levels, and interestingly these SNPs were also associated with increased cholesterol levels in the British 1958 Birth Cohort (7), which confirms that these SNPs indeed affect metabolism of lipoproteins in humans.

We found a reduced risk of COPD mortality in carriers of the T allele of rs1806649, and we thus confirm the relevance of this SNP in COPD. Canova et al. (2) have already introduced the wild-type allele (C) of rs1806649 as a factor increasing the risk of air pollution-induced asthma/COPD hospital admissions. In this light, indeed, the T allele of rs1806649 may have protective potential compared with the C allele. It would be interesting to investigate further whether this effect differs depending on smoking habits, but during 18 yr of follow-up only 20 deaths due to COPD occurred, and stratification according to smoking habits was not feasible because of a lack of study power. However, all the subjects who died of COPD were ever smokers, and we showed that the protective effect is observed in this group.

The functionally important SNP rs6721961 (−617C/A) located in the *NFE2L2* promoter region significantly impedes *NFE2L2* function and is associated with increased risk of lung cancer (20) and acute lung injury (13). In our study we tested rs4243387, which is in perfect LD (r^2^ = 1.0) with rs6721961, but we did not observe an association with mortality risk. With regard to the longevity phenotype, according to the GWAS Central database (http://www.gwascentral.org) none of the previous genome-wide association studies reported any SNP in *NFE2L2* to be associated with longevity in humans.

Polymorphisms in *NFE2L2* may alter expression of *NFE2L2* or its ability to translocate from cytoplasm to the nuclear binding sites (13). Based on the data presented by Marzec et al. (13) two functional SNPs (rs6721961 and rs6706649) impede the activity of the promoter region by >50% (indicated by lower luciferase activity in vitro) and one (rs35652124) by ∼50%. Thus in humans, we would not expect changes close to sixfold higher expression or activity, as it was shown in the naked mole rat (26). However, it is hard to predict to what extent the expression of *NFE2L2* might be altered by SNPs. It may also differ between different organs, such as lungs, liver, brain, or heart and their exposure to oxidative stress.

### Strengths and Limitations

The major strength of the current study is the longitudinal design. The follow-up period of 18 yr provided a wide time window for evaluating survival of subjects, who were sampled from the general population. Also the high follow-up rate should be mentioned, since 98.6% of the included subjects could be traced back.

We could evaluate the associations between triglyceride and cholesterol levels in males only, since these measurements were not performed in females in the Vlagtwedde-Vlaardingen cohort. Nonetheless, in the previous studies, most of the effects of *NFE2L2* on lipids levels were observed in male mice (1), and we were able to investigate the relationship, previously shown in mice, in humans.

The small number of deaths due to COPD (i.e., 20) could be considered a limitation of our study. However, SNP rs1806649, which showed association with reduced COPD mortality, was previously associated with asthma/COPD hospital admissions in the same direction (2), making our finding consistent with the results of previous studies.

In summary, this is the first study showing that *NFE2L2* plays an important role in human survival, as shown by its associations with reduced all-cause, cardiovascular, and COPD mortality in the general population.

## GRANTS

The British 1958 Birth Cohort DNA collection was funded by Medical Research Council Grant G0000934 and Wellcome Trust Grant 068545/Z/02. This work was supported by the Graduate School for Drug Exploration (GUIDE) University of Groningen, University Medical Center Groningen, the Netherlands; and the Netherlands Asthma Foundation (Grant 3.2.02.51).

## DISCLOSURES

No conflicts of interest, financial or otherwise, are declared by the author(s).

## AUTHOR CONTRIBUTIONS

Author contributions: S.M.F., J.M.V., and H.M.B. conception and design of research; S.M.F. performed experiments; S.M.F. analyzed data; S.M.F., J.M.V., and H.M.B. interpreted results of experiments; S.M.F. prepared figures; S.M.F. drafted manuscript; S.M.F., J.M.V., and H.M.B. edited and revised manuscript; S.M.F., J.M.V., and H.M.B. approved final version of manuscript.

## References

[1] Barajas B Che N Yin F Rowshanrad A Orozco LD Gong KW Wang X Castellani LW Reue K Lusis AJ Araujo JA NF-E2-related factor 2 promotes atherosclerosis by effects on plasma lipoproteins and cholesterol transport that overshadow antioxidant protection. Arterioscler Thromb Vasc Biol 31: 58–66, 2011 20947826 10.1161/ATVBAHA.110.210906 PMC3037185

[2] Canova C Dunster C Kelly FJ Minelli C Shah PL Caneja C Tumilty MK Burney P PM10-induced hospital admissions for asthma and chronic obstructive pulmonary disease: the modifying effect of individual characteristics. Epidemiology 23: 607–615, 2012 22531667 10.1097/EDE.0b013e3182572563

[3] Carnevali S Petruzzelli S Longoni B Vanacore R Barale R Cipollini M Scatena F Paggiaro P Celi A Giuntini C Cigarette smoke extract induces oxidative stress and apoptosis in human lung fibroblasts. Am J Physiol Lung Cell Mol Physiol 284: L955–L963, 2003 12547733 10.1152/ajplung.00466.2001

[4] Chorley BN Campbell MR Wang X Karaca M Sambandan D Bangura F Xue P Pi J Kleeberger SR Bell DA Identification of novel NRF2-regulated genes by ChIP-Seq: influence on retinoid X receptor alpha. Nucleic Acids Res 40: 7416–7429, 2012 22581777 10.1093/nar/gks409 PMC3424561

[5] Freigang S Ampenberger F Spohn G Heer S Shamshiev AT Kisielow J Hersberger M Yamamoto M Bachmann MF Kopf M Nrf2 is essential for cholesterol crystal-induced inflammasome activation and exacerbation of atherosclerosis. Eur J Immunol 41: 2040–2051, 2011 21484785 10.1002/eji.201041316

[6] Herskind AM McGue M Holm NV Sorensen TI Harvald B Vaupel JW The heritability of human longevity: a population-based study of 2872 Danish twin pairs born 1870–1900. Hum Genet 97: 319–323, 1996 8786073 10.1007/BF02185763

[7] Genetic information from the British 1958 Birth Cohort. http://www.b58cgene.sgul.ac.uk/, accessed 19th 11 2013

[8] Huang J Tabbi-Anneni I Gunda V Wang L Transcription factor Nrf2 regulates SHP and lipogenic gene expression in hepatic lipid metabolism. Am J Physiol Gastrointest Liver Physiol 299: G1211–G1221, 2010 20930048 10.1152/ajpgi.00322.2010 PMC3006243

[9] Hybertson BM Gao B Bose SK McCord JM Oxidative stress in health and disease: the therapeutic potential of Nrf2 activation. Mol Aspects Med 32: 234–246, 2011 22020111 10.1016/j.mam.2011.10.006

[10] Kapeta S Chondrogianni N Gonos ES Nuclear erythroid factor 2-mediated proteasome activation delays senescence in human fibroblasts. J Biol Chem 285: 8171–8184, 2010 20068043 10.1074/jbc.M109.031575 PMC2832969

[11] Lewis KN Mele J Hayes JD Buffenstein R Nrf2, a guardian of healthspan and gatekeeper of species longevity. Integr Comp Biol 50: 829–843, 2010 21031035 10.1093/icb/icq034 PMC2965188

[12] Malhotra D Thimmulappa R Navas-Acien A Sandford A Elliott M Singh A Chen L Zhuang X Hogg J Pare P Tuder RM Biswal S Decline in NRF2-regulated antioxidants in chronic obstructive pulmonary disease lungs due to loss of its positive regulator, DJ-1. Am J Respir Crit Care Med 178: 592–604, 2008 18556627 10.1164/rccm.200803-380OC PMC2542433

[13] Marzec JM Christie JD Reddy SP Jedlicka AE Vuong H Lanken PN Aplenc R Yamamoto T Yamamoto M Cho HY Kleeberger SR Functional polymorphisms in the transcription factor NRF2 in humans increase the risk of acute lung injury. FASEB J 21: 2237–2246, 2007 17384144 10.1096/fj.06-7759com

[14] Nguyen T Yang CS Pickett CB The pathways and molecular mechanisms regulating Nrf2 activation in response to chemical stress. Free Radic Biol Med 37: 433–441, 2004 15256215 10.1016/j.freeradbiomed.2004.04.033

[15] Sandford AJ Malhotra D Boezen HM Siedlinski M Postma DS Wong V Akhabir L He JQ Connett JE Anthonisen NR Pare PD Biswal S NFE2L2 pathway polymorphisms and lung function decline in chronic obstructive pulmonary disease. Physiol Genomics 44: 754–763, 2012 22693272 10.1152/physiolgenomics.00027.2012 PMC3774584

[16] Siedlinski M Postma DS Boer JM van der Steege G Schouten JP Smit HA Boezen HM Level and course of FEV1 in relation to polymorphisms in NFE2L2 and KEAP1 in the general population. Respir Res 10: 73, 2009 19671143 10.1186/1465-9921-10-73 PMC2738671

[17] Suh JH Shenvi SV Dixon BM Liu H Jaiswal AK Liu RM Hagen TM Decline in transcriptional activity of Nrf2 causes age-related loss of glutathione synthesis, which is reversible with lipoic acid. Proc Natl Acad Sci USA 101: 3381–3386, 2004 14985508 10.1073/pnas.0400282101 PMC373470

[18] Sussan TE Jun J Thimmulappa R Bedja D Antero M Gabrielson KL Polotsky VY Biswal S Disruption of Nrf2, a key inducer of antioxidant defenses, attenuates ApoE-mediated atherosclerosis in mice. PLoS One 3: e3791, 2008 19023427 10.1371/journal.pone.0003791 PMC2582492

[19] Suzuki M Betsuyaku T Ito Y Nagai K Nasuhara Y Kaga K Kondo S Nishimura M Down-regulated NF-E2-related factor 2 in pulmonary macrophages of aged smokers and patients with chronic obstructive pulmonary disease. Am J Respir Cell Mol Biol 39: 673–682, 2008 18566336 10.1165/rcmb.2007-0424OC

[20] Suzuki T Shibata T Takaya K Shiraishi K Kohno T Kunitoh H Tsuta K Furuta K Goto K Hosoda F Sakamoto H Motohashi H Yamamoto M Regulatory nexus of synthesis and degradation deciphers cellular Nrf2 expression levels. Mol Cell Biol 33: 2402–2412, 2013 23572560 10.1128/MCB.00065-13 PMC3700104

[21] Tanaka Y Aleksunes LM Yeager RL Gyamfi MA Esterly N Guo GL Klaassen CD NF-E2-related factor 2 inhibits lipid accumulation and oxidative stress in mice fed a high-fat diet. J Pharmacol Exp Ther 325: 655–664, 2008 18281592 10.1124/jpet.107.135822

[22] Tomobe K Shinozuka T Kuroiwa M Nomura Y Age-related changes of Nrf2 and phosphorylated GSK-3beta in a mouse model of accelerated aging (SAMP8). Arch Gerontol Geriatr 54: e1–e7, 2012 21784539 10.1016/j.archger.2011.06.006

[23] Ungvari Z Bailey-Downs L Gautam T Jimenez R Losonczy G Zhang C Ballabh P Recchia FA Wilkerson DC Sonntag WE Pearson K de CR Csiszar A Adaptive induction of NF-E2-related factor-2-driven antioxidant genes in endothelial cells in response to hyperglycemia. Am J Physiol Heart Circ Physiol 300: H1133–H1140, 2011 21217061 10.1152/ajpheart.00402.2010 PMC3075025

[24] Valcarcel-Ares MN Gautam T Warrington JP Bailey-Downs L Sosnowska D de Cabo R Losonczy G Sonntag WE Ungvari Z Csiszar A Disruption of Nrf2 signaling impairs angiogenic capacity of endothelial cells: implications for microvascular aging. J Gerontol A Biol Sci Med Sci 67: 821–829, 2012 22219515 10.1093/gerona/glr229 PMC3403863

[25] van Diemen CC Postma DS Vonk JM Bruinenberg M Schouten JP Boezen HM A disintegrin and metalloprotease 33 polymorphisms and lung function decline in the general population. Am J Respir Crit Care Med 172: 329–333, 2005 15879414 10.1164/rccm.200411-1486OC

[26] Yu C Li Y Holmes A Szafranski K Faulkes CG Coen CW Buffenstein R Platzer M de Magalhaes JP Church GM RNA sequencing reveals differential expression of mitochondrial and oxidation reduction genes in the long-lived naked mole-rat when compared to mice. PLoS One 6: e26729, 2011 22073188 10.1371/journal.pone.0026729 PMC3207814

[27] Zakkar M Van der Heiden K Luong LA Chaudhury H Cuhlmann S Hamdulay SS Krams R Edirisinghe I Rahman I Carlsen H Haskard DO Mason JC Evans PC Activation of Nrf2 in endothelial cells protects arteries from exhibiting a proinflammatory state. Arterioscler Thromb Vasc Biol 29: 1851–1857, 2009 19729611 10.1161/ATVBAHA.109.193375

